# Characterization of probiotics isolated from dietary supplements and evaluation of metabiotic-antibiotic combinations as promising therapeutic options against antibiotic-resistant pathogens using time-kill assay

**DOI:** 10.1186/s12906-024-04582-3

**Published:** 2024-08-14

**Authors:** Mona S. El Far, Azza S. Zakaria, Mervat A. Kassem, Eva A. Edward

**Affiliations:** Department of Microbiology and Immunology, Faculty of Pharmacy, Alexandria University, Alexandria, Egypt

**Keywords:** Probiotic dietary supplements, Metabiotics, MALDI-TOF mass spectrometry, Acid tolerance, Cell surface hydrophobicity, Autoaggregation, Phenol tolerance, Bile salt hydrolysis, Time-kill assay, Cell-free supernatants (CFS), Synergism

## Abstract

**Background:**

The global probiotics dietary supplements market size is continuously growing. To overcome probiotics’ health concerns, metabiotics are recognized as a safer alternative. Aiming to deal with the escalating antimicrobial resistance, the current work demonstrates synergistic metabiotic-antibiotic combinations against antibiotic-resistant pathogens.

**Methods:**

The probiotic properties of lactic acid bacteria (LAB) strains isolated from 3 commercial dietary supplements were characterized in vitro. The combinations of the cell-free supernatants (CFS) of selected probiotic strains and conventional antibiotics against *Staphylococcus aureus* and *Escherichia coli* clinical isolates were evaluated using the time-kill assay. To our knowledge, the current literature lacks sufficient time-kill assay studies revealing the kinetics of such metabiotic-antibiotic combinations against *S. aureus* and *E. coli*.

**Results:**

Four LAB strains isolated from dietary supplements as well as two reference strains were included in this study. The isolated LAB strains were identified by MALDI-TOF mass spectrometry as follows: P2: *Lactobacillus acidophilus,* P3: *Lactiplantibacillus plantarum*, P4: *Lacticaseibacillus rhamnosus*, and P5: *Pediococcus acidilactici.* The identification matched with that annotated by the manufacturers, except for P3. The tested strains could resist the acidic environment at pH 3. Excluding P2, the examined strains showed less than 1 log reduction in survivors upon the addition of reconstituted skimmed milk to pepsin at pH 2 and displayed an acceptable tolerance to 0.3% ox-bile. All the strains tolerated pancreatin. The hydrophobicity and autoaggregation capacities ranged between 7–92% and 36–66%, respectively. P2 was excluded owing to its inferior probiotic potential. Although the remaining strains showed excellent growth at 0.2% phenol, their growth was reduced at higher concentrations. *L. plantarum* and *P. acidilactici* strains possessed bile salt hydrolysis activity. The time-kill assay revealed promising synergistic activities of the combinations of CFS of *L. rhamnosus* P4 with either ceftazidime or gentamicin against *E. coli* and with only ceftazidime against *S. aureus*, as well as CFS of *P. acidilactici* P5 and ceftazidime against *S. aureus*.

**Conclusions:**

Strict identification and evaluation of the probiotic strains incorporated in dietary supplements is crucial to ensure their safety and efficacy. The CFS of probiotics could be utilized to formulate novel biotherapeutics targeting problematic pathogens*.* However, future in vivo studies are required to evaluate the appropriate treatment regimen.

**Supplementary Information:**

The online version contains supplementary material available at 10.1186/s12906-024-04582-3.

## Background

Owing to the noticeable escalation in the demand for functional foods in the last decades, the industry is witnessing a shooting up interest to develop probiotic dietary supplements that confer numerous health benefits [1]. The size of the global market of probiotics dietary supplements will grow from 6.94 billion USD, in 2023, to 7.53 billion USD, in 2024, and is expected to reach 10.54 billion USD in 2028 [2]. Various bacterial species are involved in the production of commercial probiotics supplements such as *Bifidobacterium, Lactobacillus, Pediococcus, Streptococcus,* and *Lactococcus* species [3].

Probiotics’ health benefits are associated with managing and preventing different diseases [4]. However, to function properly, probiotic strains should possess various criteria such as the ability to survive through the human gastrointestinal tract, gastric juices tolerance, bile salt hydrolysis, phenol tolerance as well as adhesion and colonization of the gut epithelial cells through both autoaggregation and hydrophobic properties [5–7].

The persistent development of antimicrobial resistance has posed a more pronounced impact on the population’s health worldwide. It is estimated that, globally, the death rate owing to antibiotic-resistant infections is 700,000 people annually. To overcome this disaster, researchers are forced to look for alternative non-antimicrobial therapeutic approaches, among which are probiotics. The probiotics’ antagonistic activity against antibiotic-resistant microbes could be attributed to multiple mechanisms, including the host immunity modulation, out-competing the pathogens for nutrients, occluding pathogens from the sites of adhesion, competitive exclusion via creating a hostile environment, and the production of various substances with antagonistic activity against pathogens [8].

Earlier studies have reported the antimicrobial activity exhibited by probiotics against multidrug-resistant *Staphylococcus aureus*, *Escherichia coli*, *Klebsiella pneumonia*, *Enterococcus faecalis*, *Pseudomonas aeruginosa*, *Salmonella typhii* as well as other *Salmonella* spp. [9]. Also, the probiotic-antibiotic combination successfully contributed to managing *Helicobacter pylori* infection. Besides replenishing the intestinal flora, probiotic-antibiotic combinatorial therapy provides a better antimicrobial potential, reduces the required antibiotic dose, and decreases the undesirable side effects of the antibiotics [10].

Notwithstanding that probiotics exhibit versatile health benefits; the long-term safety of their consumption is still questionable rendering their usage a “double-edged” dilemma [11]. Probiotic supplements are likely to pose a great risk of spreading antibiotic resistance determinants due to the markedly higher quantities of probiotics consumed [12]. Accordingly, to overcome the adverse effects of probiotics, metabiotics have been considered as a more preferrable alternative [13].

Metabiotics, the next-generation probiotics, are recognized as bioactive substances generated by the probiotic microorganism through its metabolic activities. They include signaling molecules, active metabolites as well as dead cells in addition to their fragments. Probiotic microorganisms can produce different types of metabiotics such as cell surface molecules, bacteriocins, short-chain fatty acids, quorum sensing molecules, and polysaccharides. Compared to traditional probiotics, metabiotics are easier for administration and more appropriate for long-term storage of functional products. Metabiotics are characterized by their anti-inflammatory, immunomodulatory, and anticancer effects as well as their potential in controlling blood pressure and reduction of the oxidative stress [14].

This study aimed to ensure that the commercial probiotic dietary supplements included in the presented work meet the requirements to deliver the intended health benefits and could be successfully used among therapeutic regimens recommended for combating antibiotic-resistant pathogens widely emerging in developing countries such as Egypt. This could be accomplished through the in vitro characterization of the probiotic properties of LAB strains isolated from 3 commercial dietary supplements belonging to different brands. In addition, the efficacy of combining the CFS of selected probiotic strains with conventional antibiotics against pathogenic clinical isolates has been evaluated.

## Methods

### Commercial probiotics and reference strains

Three commercial probiotic dietary supplements belonging to different commercial brands were included in this study. They were designated here as DSP3, DSP4, and DSP5. For the isolation of the probiotic bacterial strains from the dietary supplements, the content of one capsule was emptied in a sterile flask containing 100 mL De Man, Rogosa, Sharpe (MRS) broth (Himedia, India). In the case of DSP3, one tablet was aseptically inoculated in a 100 mL MRS broth flask. Each flask was vortexed well and incubated aerobically for 24–48 h at 37 °C [15]. Streaking on MRS agar plates was done to obtain pure cultures of the probiotic bacterial strains under investigation. Details about these dietary supplements and the isolated probiotic LAB strains are illustrated in Table 1.

**Table 1:** Information on dietary supplements and the isolated probiotic LAB strains

**Dietary supplement code**	**Country of the manufacturer**	**Dosage form**	**Probiotic content as claimed on the label**	**Count/tablet or capsule as claimed on the label**	**Code of isolated LAB strain**	**Probiotic LAB strain as identified by MALDI**			
						Organism (best match)	MALDI score value^a^	Organism (second best match)	MALDI score value^a^
**DSP3**	USA	Tablet	*Lactobacillus acidophilus*	100 million CFU	P2	*Lactobacillus acidophilus*	2.184	*Lactobacillus acidophilus*	2.16
					P3	*Lactiplantibacillus plantarum*	2.352	*Lactiplantibacillus plantarum*	2.35
**DSP4**	USA	Capsule	*Lacticaseibacillus rhamnosus*	20+ billion CFU	P4	*Lacticaseibacillus rhamnosus*	2.485	*Lacticaseibacillus casei*	2.341
**DSP5**	USA	Capsule	*Pediococcus acidilactici*	4 billion CFU	P5	*Pediococcus acidilactici*	2.07	*Pediococcus acidilactici*	1.988
			*Saccharomyces boulardii*						

^a^MALDI score value of the organism’s first and second-best match scores. MALDI score value ranging between 2.300 - 3.000 indicates highly probable species identification, while a score ranging between 1.700 - 1.999 indicates probable genus identification

In addition, two reference LAB strains, *Pediococcus acidilactici* ATCC 8042 and *Lactiplantibacillus plantarum* ATCC 8014, were included in the tests for the phenotypic identification and in vitro characterization of probiotic potential, along with the tested isolates, as controls. They were purchased from the Microbiological Resources Center (Cairo MIRCEN, Egypt), and were given the codes Ref 1 and Ref 2, respectively.

### Screening and identification of LAB isolates

For the detection of acid-producing bacteria, an overnight culture of the tested isolates was streaked onto MRS agar plates containing 1% CaCO_3_ (Adwic, Egypt), then incubated aerobically at 37 °C for 24–48 h. Acid-producing bacteria were detected by the presence of a clear zone surrounding the colony due to the dissolution of CaCO_3_ [16, 17]. Acid-producing bacterial strains were then identified through various morphological, biochemical, and physiological tests. The bacterial strains were subjected to Gram stain, endospore stain, catalase test, and motility test [17–19].

The gas production test was used to investigate the homofermentative and heterofermentative properties of the LAB isolates. One percent of an overnight culture of each isolate was inoculated in test tubes containing the modified sterile MRS broth (containing ammonium sulfate instead of ammonium citrate) and Durham’s tube, then incubated at 37 °C for 24 h. Displacement of the liquid in Durham’s tube by CO_2_ gas, produced from the fermented glucose, was considered as a positive result [20]. Regarding the arginine hydrolysis test, one percent of an overnight culture of each of the tested isolates was inoculated in the sterile modified MRS broth containing 0.3% (w/v) arginine (Nice chemicals Co., India), and 0.05% glucose (without the addition of beef extract). After 5 days of aerobic incubation at 37 °C, a few drops of Nessler’s reagent were added. A positive reaction was obtained from the production of ammonia due to the hydrolysis of arginine [20, 21]. The bright orange color indicated a positive reaction, while the yellow color indicated a negative reaction. A negative control (modified MRS broth without arginine) was included in the experiment [22].

The isolates were tested for their ability to grow at different NaCI concentrations. Overnight cultures of LAB isolates were 1% inoculated into MRS broth with (2%, 4%, 6.5%, and 8% w/v) NaCl or without NaCl (as a positive control) in a 96-well microtiter plate. After 24–48 h of incubation at 37 °C, the absorbance at 630 nm was measured for each strain and 3 measurements were considered [23]. The percentage of growth inhibition of the tested isolates at different NaCl concentrations was calculated as follows:

$$\mathrm{Percentage}\;\mathrm{inhibition}\;=\frac{({\mathrm{OD}}_{630\;}\mathrm{of}\;\mathrm{the}\;\mathrm{positive}\;\mathrm{control}\ {\text -}\ {\mathrm{OD}}_{630}\;\mathrm{of}\;\mathrm{test}\;\mathrm{sample})}{{\mathrm{OD}}_{630}\;\mathrm{of}\;\mathrm{the}\;\mathrm{positive}\;\mathrm{control}}\text X\ 100.$$

Data were expressed as means ± S.D.

To investigate the capability of the tested isolates to grow at various temperatures, the growth of each isolate in 5 mL MRS broth was examined after incubation at 15 °C for 14 days and at 45 °C for 7 days [24].

For further identification of the recovered probiotic strains to the species level, they were proteomically analyzed through MALDI-TOF MS using MALDI Biotyper (Bruker Daltonik, USA) [25].

### In vitro characterization of probiotic properties

#### Survival under conditions simulating the human gastrointestinal tract (GIT)

Bacterial cells from 5 mL overnight cultures were harvested by centrifugation (7000 rpm, 10 min, 4 °C), washed twice with phosphate-buffered saline (PBS) (pH 7.2), then subjected to the following stress conditions simulating those encountered in the GIT [26]:

##### Acid and pepsin tolerance

To examine the isolates’ survival at low pH, the washed bacterial cells were resuspended in 1 mL PBS solution with a pH 3 using 1 M HCl. To test for pepsin tolerance, the bacterial pellets were resuspended in 1 mL PBS solution adjusted to pH 3 or pH 2, containing pepsin (Oxford Co., India) (3 mg/mL). Incubation was done at 37°C for 3 h [26].

##### Investigating the effect of reconstituted skimmed milk (RSM) in simulated gastric juice on bacterial tolerance

To mimic the in vivo gastric digestion conditions, RSM (11% solids, w/v) was added to a PBS solution of pH 2 to reach a final pH of approximately 3, and then pepsin (3 mg/mL) was added. The washed bacterial cells were resuspended in such solution and incubated for 3 h at 37 °C [26, 27].

##### Pancreatin and bile tolerance

To examine the tolerance of the tested isolates to pancreatin or bile, the washed bacterial pellets were resuspended in 1 mL PBS solution (pH 8) containing 1mg/mL pancreatin (LOBA Chem, India), or 0.3% ox-bile (LOBA Chem, India), respectively, then incubated at 37°C for 4 h [26, 28, 29].

For all tests, the bacterial tolerance was assessed in terms of viable colony counts. Resistant strains were those whose initial counts did not decline by greater than one log CFU/mL after the designated incubation period [30]. The percentage of survivors was calculated for each strain and data were expressed as means ± S.D.

#### Adhesion activity detection

##### Cell surface hydrophobicity (CSH)

CSH of the tested isolates was determined by measuring the affinity of an overnight bacterial cell culture to xylene in a two-phase system. For each strain, the bacterial culture was harvested by centrifugation at 7000 rpm for 10 min at 4 °C, washed twice in sterile PBS (pH 7), and resuspended in PBS to an OD_630_ of approximately 0.6. One milliliter of xylene (Adwic, Egypt) was added to a test tube of 3 mL of the washed cells. The tube of the tested strain was vortexed for 2 min and the suspensions were left for 30 min to allow phase separation. Aliquots were drawn from the aqueous phase to measure OD_630_ in a 96-well microtiter plate for each strain and 3 measurements were considered. The percentage cell surface hydrophobicity was calculated as:

$${\text{Hydrophobicity}}\;\%\;=\frac{({\text{OD}}_{630\;}\text{before}\;\text{adding}\;\text{xylene}\ {\text-}\ {\text{OD}}_{630}\;\text{after}\;\text{adding}\;\text{xylene})}{({\text{OD}}_{630}\;\text{before}\;\text{adding}\;\text{xylene})}\text X\;100.\\$$

The strains were classified into 3 groups: those with high hydrophobicity (71–100%), intermediate hydrophobicity (36–70%), and low hydrophobicity (0–35%) [31]. Data were expressed as means ± S.D.

##### Autoaggregation activity

LAB strains were aerobically grown overnight in MRS broth at 37 °C. For each strain, the bacterial culture was centrifuged at 7000 rpm for 10 min at 4 °C, and the bacterial pellet was then resuspended in 4 mL of PBS (pH 7) to about 10^8^ CFU/mL (OD_630_ 0.2–0.3). The suspension of the tested bacterial strain was then vortexed for 10 s and incubated at room temperature for 5 h. Aliquots of 200 µL were withdrawn from the upper part of the bacterial suspension at 0 h and 5 h intervals to measure the absorbance at 630 nm in a 96-well microtiter plate for each strain and 3 measurements were considered. The autoaggregation percentage was calculated as follows:

$$\mathrm{Autoaggregation}\;(\%)\;=1\;-({\mathrm A}_{\mathrm t}/{\mathrm A}_0)\;\mathrm x\;100.$$

Where A_0_ and A_t_ are the absorbance values at t = 0 h and 5 h, respectively [32]. Data were expressed as means ± S.D.

#### Additional tests for examining some desirable probiotic properties among the selected LAB strains

##### Phenol tolerance

One percent of overnight cultures of the selected isolates were inoculated into MRS broth with (0.2%, 0.3%, 0.4%, and 0.5% v/v) phenol (Prolabo, France) or without phenol (as a positive control) in a 96-well microtiter plate. After 24–48 h of incubation at 37 °C, the absorbance at 630 nm was measured for each strain and 3 measurements were considered. The percentage of growth of the tested isolates at different phenol concentrations was calculated as follows [33]:

$$\mathrm{Percentage}\,\mathrm{of}\,\mathrm{growth}=\frac{{\mathrm{OD}}_{630}\;\mathrm{of}\,\;\mathrm{the}\;\mathrm{test}\;\,\mathrm{sample}}{{\mathrm{OD}}_{630}\;\mathrm{of}\;\,\mathrm{the}\,\;\mathrm{positive}\;\,\mathrm{control}}\text{X}\;100.$$

Data were expressed as means ± S.D.

##### Bile salt hydrolysis (BSH) test

MRS agar, containing 0.3% w/v ox-bile and 0.37 g/L CaCl_2_ (Fischer Scientific, USA), was used. A volume of 10 µL of the overnight culture of each strain was spotted on a sterile MRS medium, then the plates were aerobically incubated at 37 °C for 24 h. Visible halos with their variable diameters around the inoculum indicated the positive BSH activity of the strains. Potential probiotic strains grown on MRS agar without ox-bile were included in the experiment as a negative control [34].

### Time-kill assay of the CFS of selected probiotic candidates combined with conventional antibiotics against pathogenic clinical isolates

For the preparation of the CFS of the tested probiotic strains, each strain was propagated in 30 mL MRS broth (pH 6.5) for 48 h at 37 °C. CFS was obtained by centrifuging the culture twice at 10,000 rpm for 15 min at 4 °C, and then the supernatant was filtered through a cellulose acetate syringe filter (Filter-bio Co., China) of 0.22 μm pore size [35].

The antibacterial activity of the CFS of 2 selected probiotic strains: *L. rhamnosus* P4 *and P. acidilactici* P5, as representatives from different genera, was assessed in combination with each of gentamicin, ciprofloxacin, and ceftazidime against one *E. coli* clinical isolate from the GIT (*E. coli*^GIT^), one *E. coli* (*E. coli*^UTI^) and one *S. aureus* (*S. aureus*^UTI2^) clinical isolates causing urinary tract infections (UTIs) using the time-kill assay.

The determination of the minimum inhibitory concentration (MIC) values of the tested antibiotics as well as the CFS of *L. rhamnosus* P4 *and P. acidilactici* P5 against the tested clinical isolates was done using the broth microdilution technique [36–38]. The solutions of each of the antibiotics and CFS of each probiotic were prepared to reach final concentrations equivalent to ¼ MIC of each antibiotic or probiotic against the organism under test. Aliquots of 5 mL sterile double-strength nutrient broth, distributed in 50 mL sterile falcons, received the required volumes of antibiotic, probiotic, and the organism under test. The reaction mixtures were carried out as follows: the first mixture received a single antibiotic component, the second received the CFS of the tested probiotic, the third received both agents (the antibiotic and CFS of the tested probiotic), and the fourth one received only sterile distilled water as the control. The final volume in all falcons was 10 mL. An overnight culture of each clinical isolate was added to each reaction mixture to reach a final count of approximately 10^6^ CFU/mL.

The cultures were incubated at 37°C with continuous shaking at 150 rpm. Samples, of 100 µL volume, were aseptically withdrawn at 0, 3, 6 and 24 h, ten-fold serially diluted and then dropped onto sterile nutrient agar plates. After overnight incubation, the number of colonies/sectors was calculated. For each clinical isolate, the log number of survivors per mL, before and after treatment, was plotted against the killing time. In addition, the antibacterial effect of the CFS of the tested probiotic as well as the selected antibiotic on *S. aureus* or *E. coli* isolates, at the same applied concentrations, was also investigated and plotted [37, 39]. Data were expressed as means ± S.D. Interpretation of the results was done according to Chambers and Sande [40] as follows:

Synergism was defined as ≥ 2 log decrease in survivors, antagonism was interpreted as > 2 log increase in survivors, and additivity was designated as ≤ 1 log change (increase or decrease) in killing when comparing the combination with the most active single component alone at any point along the time-kill curve.

## Results

### Isolation, screening and identification of LAB isolates from commercial dietary supplements

Besides the two reference LAB strains, *P. acidilactici* ATCC 8042 and *L. plantarum* ATCC 8014, adopted in this study, four probiotic bacterial strains were isolated from the three tested commercial probiotic dietary supplements. These probiotic strains were designated as follows: P2 and P3 (from DSP3), P4 (from DSP4), and P5 (from DSP5) **(**Table 1**)**. The tested strains were acid producers manifested by the detection of clear zones surrounding the colonies in the CaCO_3_ test. All were Gram-positive, non-endospore forming, catalase-negative, and non-motile bacteria. When examined microscopically, Ref 1 and P5 were cocci while Ref 2, P2, P3, and P4 appeared as rods.

After confirming these typical characteristics of LAB, the tested isolates were subjected to further investigation using biochemical and physiological tests. The tested isolates, along with the reference LAB strains, did not produce CO_2_ gas, so they were recognized as homofermentative LAB strains. Regarding the arginine hydrolysis test, Ref 1 and P5 hydrolyzed arginine producing ammonia as manifested by the appearance of the bright orange color upon the addition of Nessler’s reagent. However, Ref 2, P2, P3 and P4 didn’t produce ammonia and gave a yellow color when the reagent was added. When tested for their growth patterns at different temperatures, the tested isolates and both reference strains grew upon cultivation at both 45 °C and 15 °C, except for P2 which only survived at 45 °C while it did not show growth throughout the 7 days incubation period at 15 °C.

**Fig. 1:**
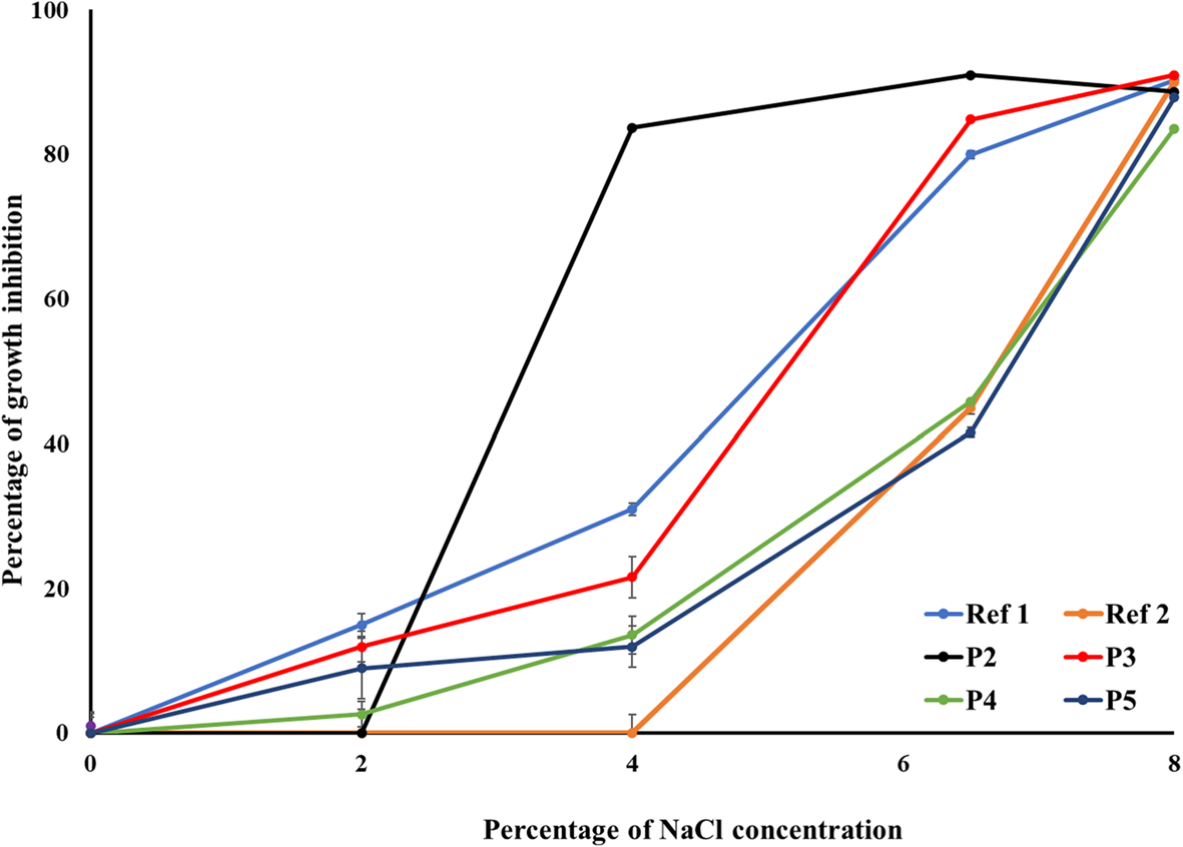
Percentage of growth inhibition of the reference LAB strains and isolates obtained from dietary supplements at different NaCl concentrations

The effect of different NaCl concentrations (2%, 4%, 6.5%, and 8%) on the growth of the tested isolates as well as the reference LAB strains was assessed. Reference LAB strains showed an appropriate growth at 2% and 4% NaCl. The growth of Ref 1 was inhibited by only 14% and 30% at 2% and 4% NaCl, respectively, while such NaCl concentrations did not affect the growth of Ref 2. Nonetheless, the growth of Ref 1 and Ref 2 was markedly inhibited by 6.5% NaCl with about 79% and 44% inhibition, respectively, while their growth was maximally suppressed at 8% NaCl concentration reaching up to approximately 90% inhibition. Similarly, isolates obtained from dietary supplements grew well at 2% with an average of 5% growth inhibition. Additionally, P3 and P5 showed adequate growth at 4% NaCl with percentages of growth inhibition ranging between 11% (in the case of P5) and 22% (in the case of P3). On the other hand, the growth of P2 was suppressed by 84% at the same NaCl concentration. The growth of the isolates declined at 6.5% NaCl where the least percentage of growth inhibition was for P5 (42%), while the maximal inhibition was observed in the case of P2 (88%). All the tested isolates showed minimal growth at 8% NaCl concentration with an average ca. 88% growth inhibition. However, the growth of the isolates, P2 and P3, was not greatly affected at 8% NaCl, compared to the obtained growth at 6.5% NaCl (Fig. 1).

For further identification of the recovered LAB strains to the species level, they were proteomically analyzed through MALDI-TOF MS. Three out of the 4 tested LAB strains belonged to *Lactobacillus* spp. and were identified as follows: P2: *Lactobacillus acidophilus*, P3: *Lactiplantibacillus plantarum*, and P4: *Lacticaseibacillus rhamnosus*. The remaining probiotic strain P5 was identified as *Pediococcus acidilactici*. The results of the identification of the isolated LAB strains using the MALDI-TOF mass spectrometry are listed in Table 1.

### In vitro characterization of probiotic properties

#### Survival under conditions simulating the human GIT

**Fig. 2:**
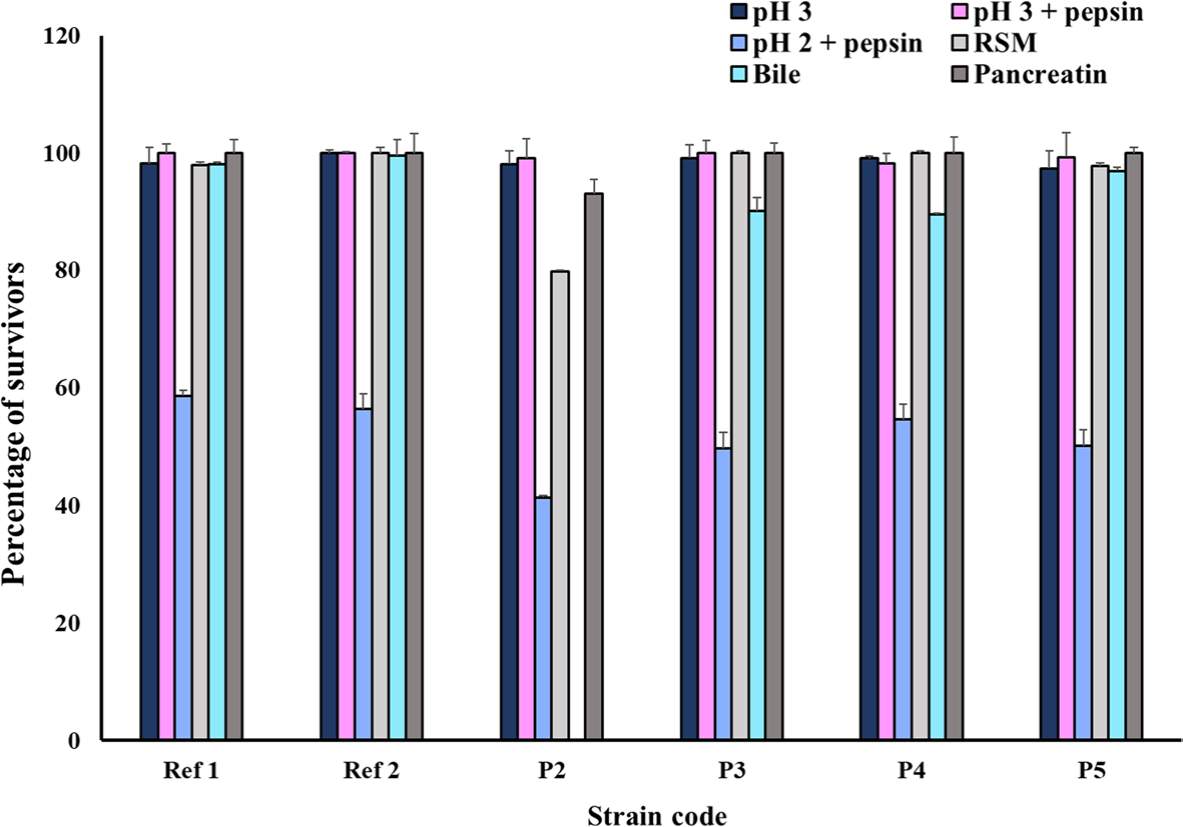
Percentage of survivors of reference LAB strains and isolates obtained from dietary supplements under conditions simulating the GIT. RSM: Reconstituted skimmed milk in the presence of pepsin at pH 2

The reference LAB strains as well as the tested isolates from dietary supplements showed less than one log reduction in the number of survivors when placed in a PBS solution of pH 3. Upon addition of pepsin to such media, and after 3 h of incubation, there was no obvious change in the bacterial count compared to their initial count at 0 h. On the contrary, there was a remarkable reduction in the viable count of the tested strains when exposed to pepsin at pH 2, with log reduction in survivors ranging between 4.2 and 5.2. However, the examined LAB strains showed better survival upon the addition of RSM to pepsin at pH 2 (< 1 log reduction in survivors), except for isolate P2 which showed a 1.66 log reduction in survivors. After 4 h of incubation of the tested strains with 0.3% ox-bile, at pH 8, most of them displayed an acceptable tolerance with log reduction in survivors ranging between 0.05 and 1. However, the survival of P2 was grossly affected by such conditions showing greater than 7 log reduction in survivors. Nonetheless, all the tested strains showed an acceptable tolerance (< 1 log reduction in survivors) to pancreatin. The percentage of survivors of reference LAB strains and the isolates obtained from dietary supplements under conditions simulating the GIT is illustrated in Fig. 2.

#### Adhesion activity detection

**Fig. 3:**
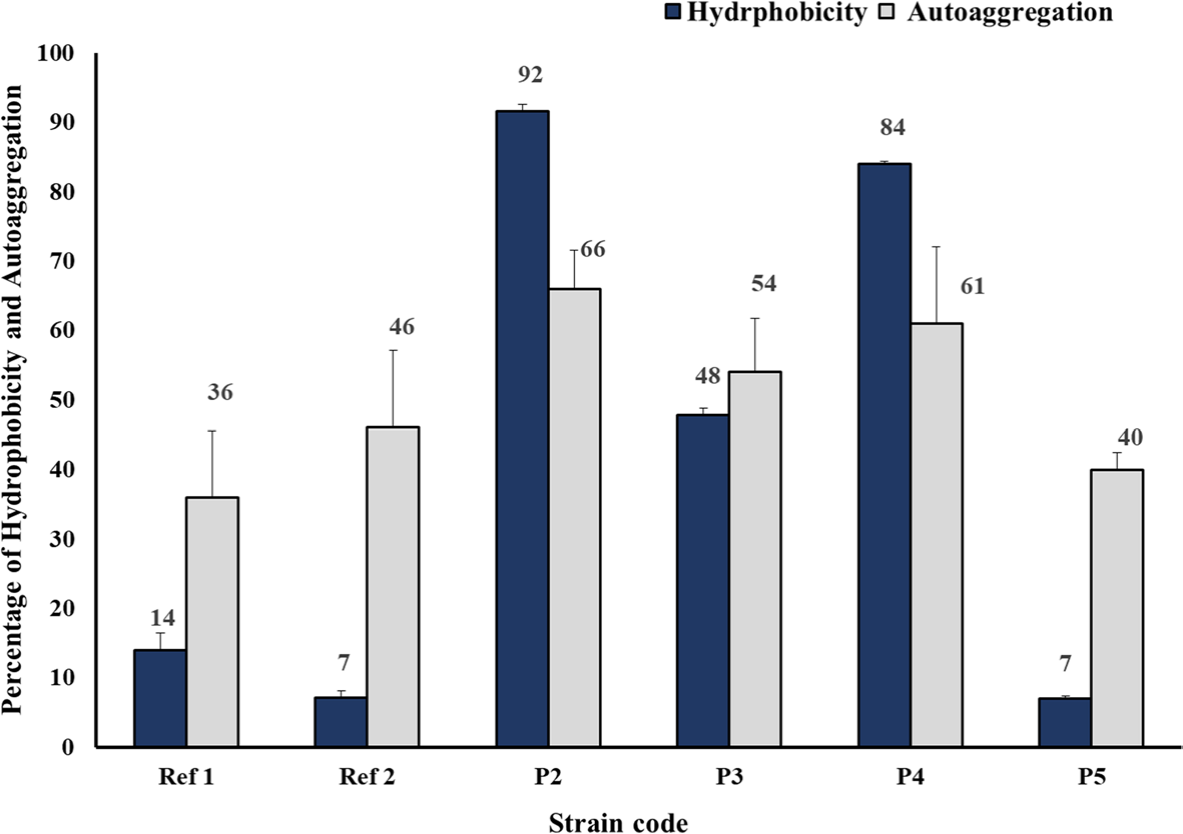
Percentage of cell surface hydrophobicity and autoaggregation among the reference LAB strains and the isolates obtained from dietary supplements

Figure 3 represents the tested strains’ CSH and autoaggregation potential. Both reference LAB strains showed low hydrophobicity percentages (14% and 7% for Ref 1 and Ref 2, respectively). Regarding LAB isolates recovered from commercial products, both strains *L. acidophilus* P2 and *L. rhamnosus* P4 showed high hydrophobicity potential of 92 and 84%, respectively. *L. plantarum* P3 showed an intermediate hydrophobicity capacity (48%), while *P. acidilactici* P5 displayed the least hydrophobicity (7%). The autoaggregation values of the reference LAB strains were about 36% and 46% for Ref 1 and Ref 2, respectively. The LAB strains isolated from dietary supplements displayed autoaggregation capacities ranging between 40% (in the case of *P. acidilactici* P5) and 66% (for *L. acidophilus* P2). Comparable autoaggregation capabilities were recorded for both P3 and P4 (54 and 61% of autoaggregation, respectively).

Based on the previous results, 3 LAB strains isolated from dietary supplements (*L. plantarum* P3, *L. rhamnosus* P4, and *P. acidilactici* P5) were considered to be potential probiotic candidates, and were included in the rest of the experiments together with the reference LAB strains. *L. acidophilus* P2 was excluded as it showed the least survival potential upon the addition of RSM to pepsin at pH 2. Also, it could not survive in the presence of 0.3% ox-bile.

### Additional tests for examining some desirable probiotic properties among the selected LAB strains

#### Phenol tolerance

**Fig. 4:**
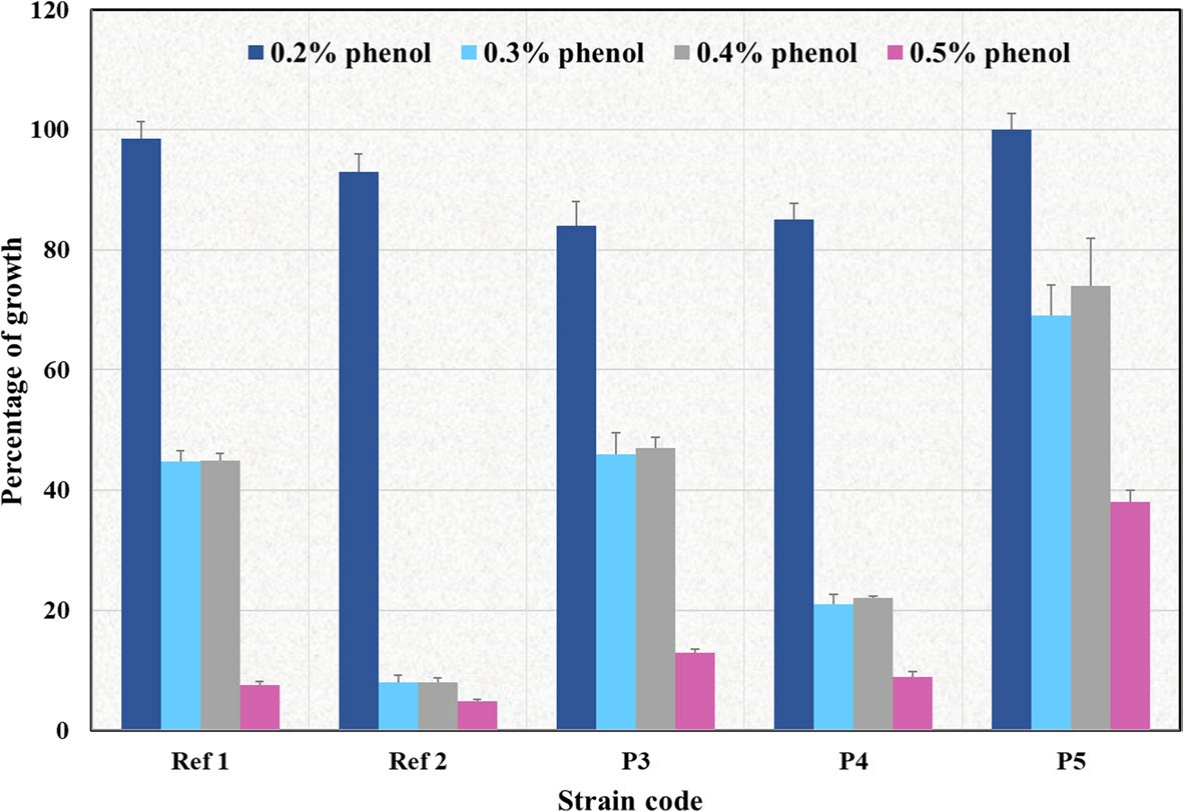
Percentage of growth of reference LAB strains and the probiotic candidates isolated from dietary supplements at different phenol concentrations

The reference LAB strains grew well in the presence of 0.2% phenol with percentages of relative growth exceeding 90%. However, there was a considerable inhibition of their growth at 0.3% phenol showing percentages of relative growth of 45% and 7% for Ref 1 and Ref 2, respectively. Additionally, the growth was maximally inhibited at 0.5% phenol with minimal percentages of relative growth noticed for both strains. Similarly, the 3 probiotic strains P3, P4, and P5 showed adequate growth at 0.2% phenol with percentages of relative growth ranging between 84% (for P3 and P4) and 100% (in the case of P5). Nonetheless, the growth was markedly affected by 0.3% phenol where the minimum percentage of relative growth (20%) was for P4, while P5 was the least affected with 72% of relative growth. Moreover, the maximum growth inhibition for the 3 isolates was observed at 0.5% phenol with percentages of relative growth ranging between 9% (in the case of P4) and 39% (in the case of P5). Notably, the growth did not greatly vary at 0.4% phenol, for any of the tested strains, compared to 0.3% phenol (Fig. 4).

#### Bile salt hydrolysis (BSH) test

Zones of precipitation were obvious around BSH-positive LAB isolates (P3 & P5) as well as the reference LAB strains. However, the diameters of the halos varied among the strains ranging between 11 and 22 mm. BSH-positive LAB strains mainly belonged to *L. plantarum* (Ref 2, and P3) and *P. acidilactici* (Ref 1 & P5). On the other hand, P4 was considered a BSH-negative strain showing no halo around the inoculated spot.

### Time-kill assay of the CFS of selected probiotic candidates combined with conventional antibiotics against pathogenic clinical isolates

#### Combinations of CFS of *L. rhamnosus* P4 with different antibiotics

**Fig. 5:**
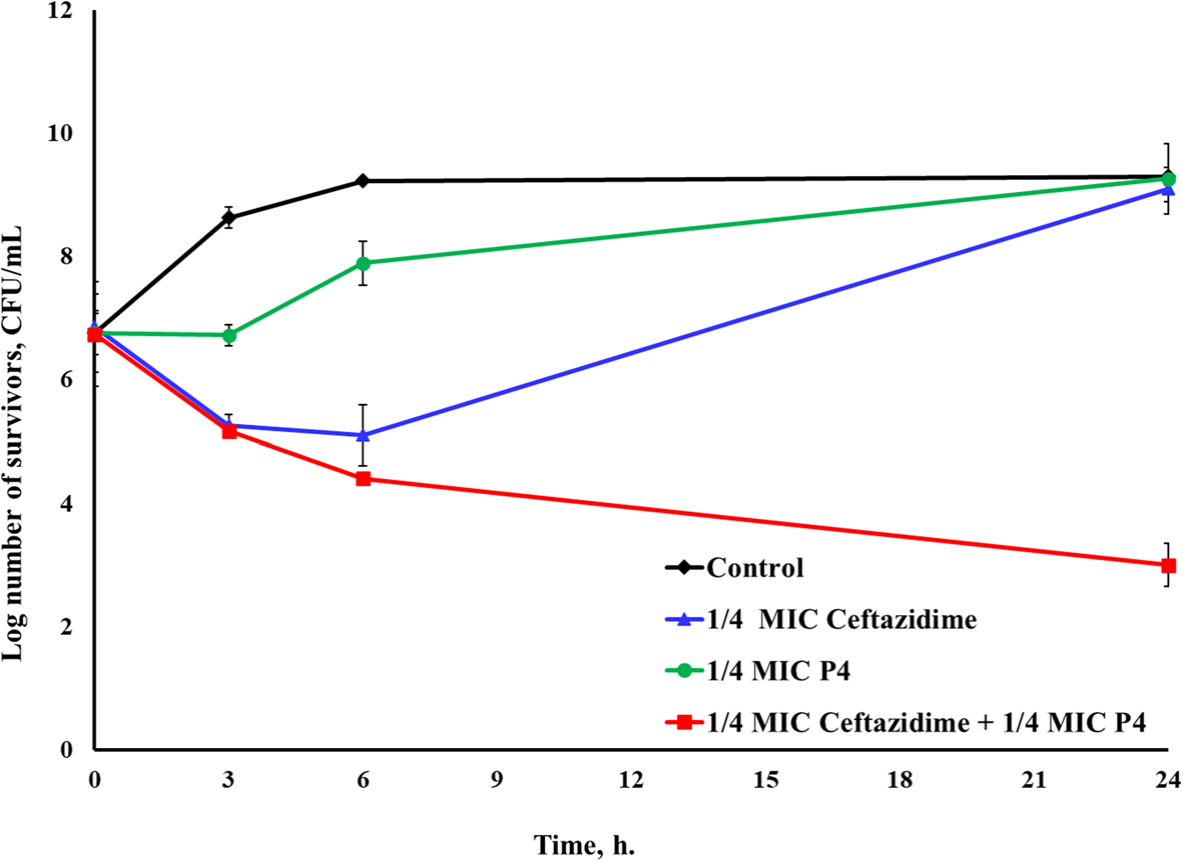
Time-kill assay of the CFS of *L. rhamnosus* P4 and ceftazidime, each alone and in combination, against *E. coli*^GIT^

**Fig. 6:**
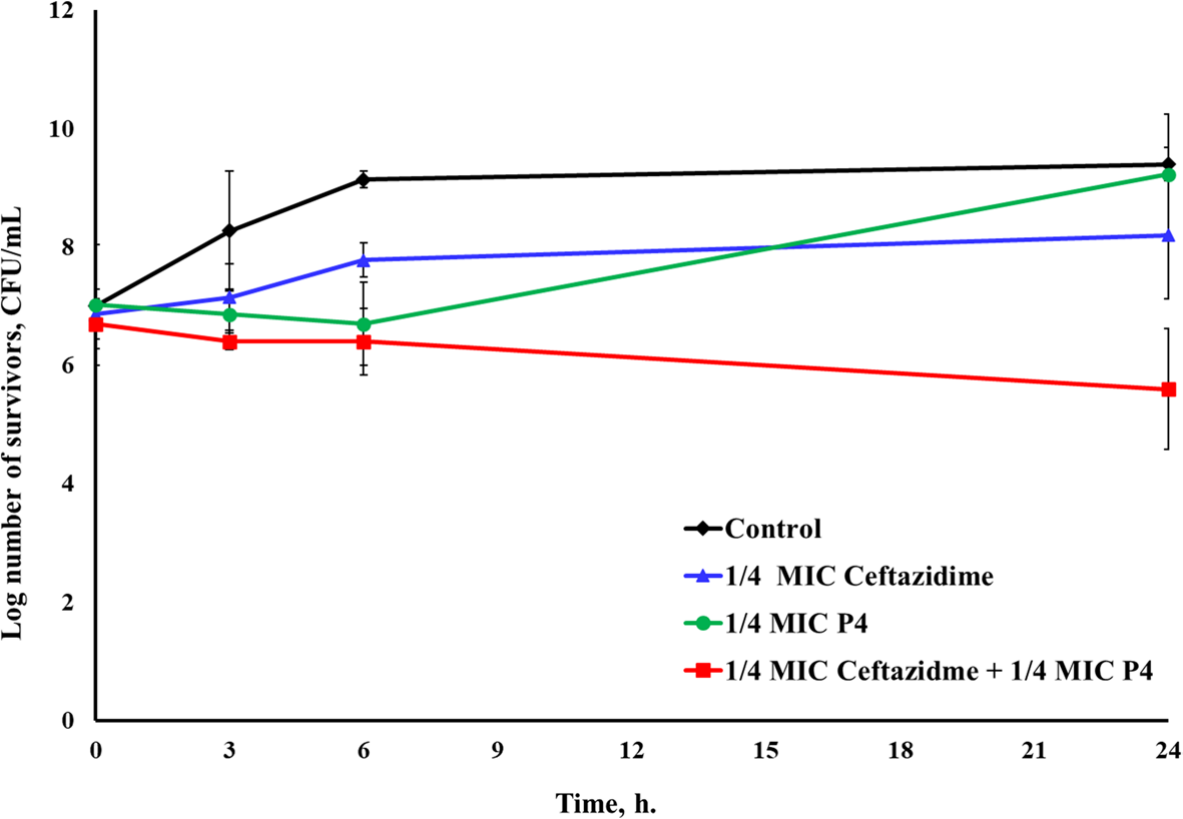
Time-kill assay of the CFS of *L. rhamnosus* P4 and ceftazidime, each alone and in combination, against *S. aureus*^UTI2^

In the case of P4/ceftazidime combination, additive effects were attained at 3 and 6-h intervals against the selected pathogenic clinical isolates *E. coli*^GIT^, *E. coli*^UTI^, and *S. aureus*^UTI2^. After 24 h, a slight additive effect was still observed against *E. coli*^UTI^. However, noticeable synergistic actions of the combination were obtained against *E. coli*^GIT^ and *S. aureus*^UTI2^ with 6.1 and 2.6 log reduction in survivors, respectively (Additional file 1) (Figs. 5 and 6).

**Fig. 7:**
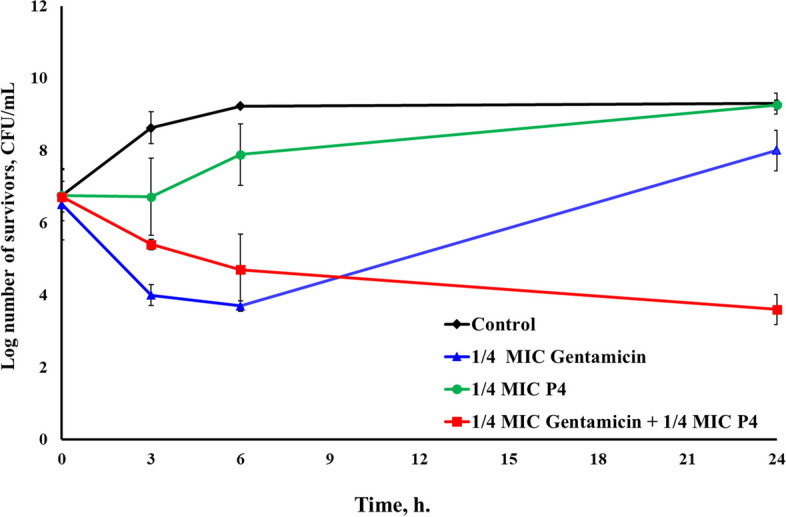
Time-kill assay of the CFS of *L. rhamnosus* P4 and gentamicin, each alone and in combination, against *E. coli*^GIT^

Regarding P4/gentamicin combination, antagonistic actions were recorded against *E. coli*^UTI^, after 3 and 6 h with about 2.16 and 2.54 log increase in survivors, respectively. However, additivity was noticed against *E. coli*^GIT^ and *S. aureus*^UTI2^ at the same contact times. After 24 h, additive effects were also observed against *E. coli*^UTI^ and *S. aureus*^UTI2^. On the other hand, a remarkable synergism was noticed against *E. coli*^GIT^ with a 4.4 log reduction in survivors (Additional file 2) (Fig. 7).

At 3 and 6-h intervals, antagonism was noticed in the case of P4/ciprofloxacin combination against *E. coli*^GIT^ with about 3.18 and 4.59 log increase in survivors, respectively. On the other hand, additivity was attained against *E. coli*^UTI^ and *S. aureus*^UTI2^ at the same time intervals. Such a combination also yielded additive effects against the three clinical isolates after 24 h (Additional file 3).

#### Combinations of CFS of *P. acidilactici* P5 with different antibiotics

**Fig. 8:**
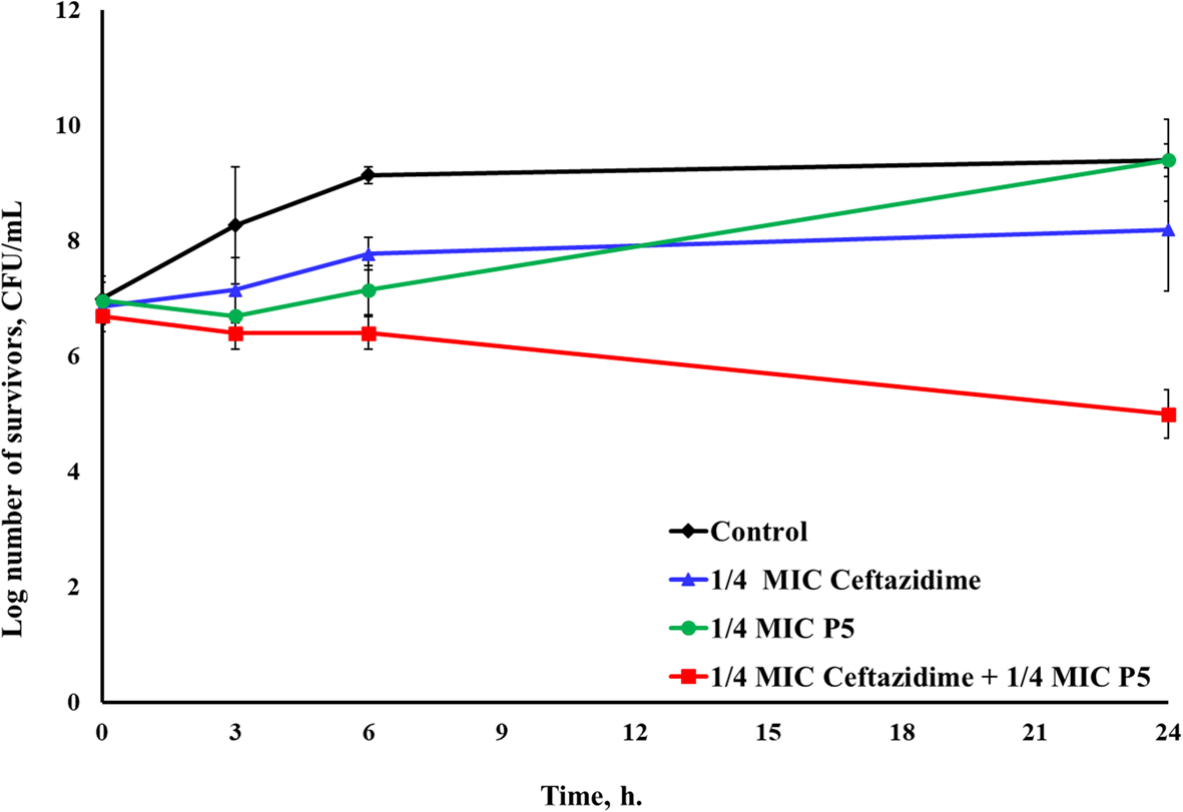
Time-kill assay of the CFS of *P. acidilactici* P5 and ceftazidime, each alone and in combination, against *S. aureus*^UTI2^

P5/ceftazidime combination yielded additive effects against *E. coli*^GIT^, *E. coli*^UTI^, and *S. aureus*^UTI2^ at 3 and 6-h intervals. After 24 h, additivity was still noticed against *E. coli*^GIT^ and *E. coli*^UTI^. On the contrary, a pronounced synergism was attained against *S. aureus*^UTI2^ with a 3.2 log reduction in survivors (Additional file 4) (Fig. 8).

At 3 and 6-h intervals, antagonistic effects were noticed in the case of P5/gentamicin combination against *E. coli*^GIT^ with about 2.2 and 2.4 log increase in survivors, respectively. On the other hand, the same combination resulted in additive actions against *E. coli*^UTI^ and *S. aureus*^UTI2^ at 3 and 6-h contact times. After 24 h, additivity was also obtained against the three pathogens (Additional file 5).

In the case of P5/ciprofloxacin combination, against *E. coli*^GIT^, antagonism was detected with about 2.76 and 3.09 log increase in survivors at 3 and 6-h intervals, respectively. However, such combination yielded additive actions against *E. coli*^UTI^ and *S. aureus*^UTI2^ at the same time intervals. Additivity was also observed against the tested clinical isolates at 24-h contact time (Additional file 6).

**Fig. 9:**
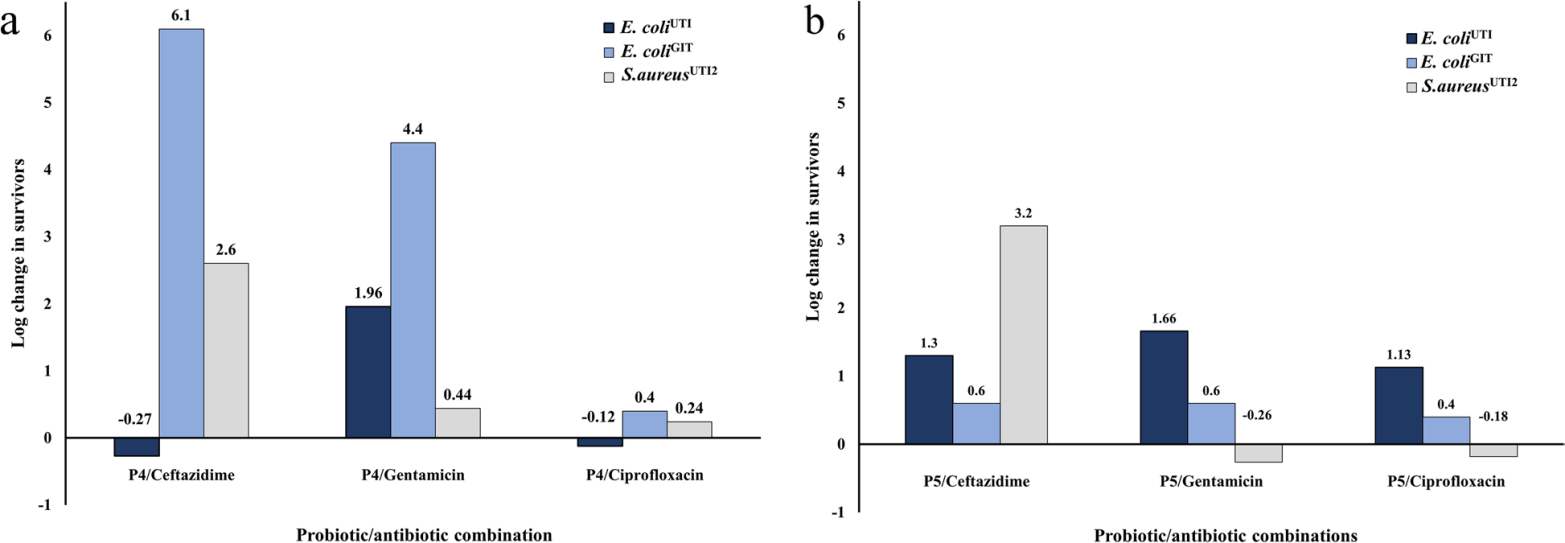
Log change in survivors (as measured by colony counts) of 3 pathogenic clinical isolates resulting from the combination of ¼ MIC of (**a**) *L. rhamnosus* P4 and (**b**) *P. acidilactici* P5 with each of ceftazidime, gentamicin, and ciprofloxacin using the time-kill assay at 24-h interval. Additivity was defined as ≤ 1 log change (increase or decrease) in killing when comparing the combination with the most active single component alone. Synergism was designated as ≥ 2 log decrease in survivors compared to the most active single component alone

Log change in survivors of the three tested pathogenic clinical isolates resulting from the combination of *L. rhamnosus* P4 and *P. acidilactici* P5 with each of ceftazidime, gentamicin, and ciprofloxacin, using the time-kill assay, at 24-h interval, is illustrated in Fig. 9a & b, respectively.

## Discussion

Probiotics have been recognized as outstanding functional foods and nutraceuticals which grabbed remarkable attention in the pharmaceutical and food sectors. Owing to their biotherapeutic impact and numerous health-beneficial effects, probiotic-based products have charmed the consumers [41]. Among various food categories, probiotic supplements constitute the greatest number of probiotics that people consume nowadays due to the billions of diverse probiotic bacteria contained in each serving [12].

In the present study, 4 LAB strains were isolated from 3 commercial dietary supplements. In addition, 2 reference LAB strains, *P. acidilactici* ATCC 8042 and *L. plantarum* ATCC 8014, were included in this study to rely on them as controls in the phenotypic identification and in vitro probiotic characterization tests. They are well-established probiotic strains that have been adopted as reference strains in previous studies [42–44]. The collected isolates were initially screened for acid-producing strains using CaCO_3_. When CaCO_3_ is added to the MRS medium, it reacts with lactic acid produced by LAB and forms the soluble calcium lactate which is manifested by the formation of clear zones around the colonies [45]. The 4 isolated strains from commercial products belonged to *Lactobacillus and Pediococcus* genera. Most of the biochemical and physiological results of the identified LAB strains were in agreement with what was published in the literature [46, 47]. The test for gas production from glucose is a key test to distinguish between homo- and heterofermentative LAB [47]. All the tested isolates did not produce gas, along with the reference LAB strains, so they were generally recognized as homofermentative LAB strains [48, 49].

Arginine dihydrolase system (ADS) can be considered as one of the neutralization mechanisms that help in the acid resistance of LAB. Generally, ADS contains 3 enzymes in LAB: arginine dihydrolase, carbamate kinase, and ornithine transcarbamylase. Arginine dihydrolase catalyzes the hydrolysis of arginine into ornithine, carbon dioxide, and ammonia. This may result in the neutralization of acids and contribute to maintaining the pH homeostasis among other mechanisms in LAB [50]. The arginine hydrolysis test and production of ammonia can be used as a biochemical test for the differentiation between various species. In our study, only Ref 1 and P5 produced ammonia from arginine hydrolysis while the rest of the strains were negative for arginine dihydrolase activity.

Generally, LAB have a good NaCl tolerance. LAB cells lose their turgor pressure when grown in a high salt medium, subsequently influencing their metabolism. Hence, they exhibit adaptive mechanisms to overcome the increased osmotic potential by pressure regulation in the internal and external cells’ environment. Moreover, good salt tolerance helps LAB in their metabolic activities, which results in acid production that further suppresses the proliferation of harmful organisms [51]. In our study, all the tested strains showed significant growth at 4% NaCl with percentages of growth inhibition ranging between 0% (in the case of Ref 2) and 30% (in the case of Ref 1). However, the growth of P2 was suppressed by 84% at the same NaCl concentration. The growth of the tested strains generally declined at higher NaCl concentrations.

When proteomically analyzed through MALDI-TOF MS, the isolated LAB strains were identified as follows: P2: *Lactobacillus acidophilus,* P3: *Lactiplantibacillus plantarum*, P4: *Lacticaseibacillus rhamnosus,* and P5: *Pediococcus acidilactici.* The identification of the isolated strains matched with that annotated by the dietary supplements’ manufacturers, except for DSP3 where multispecies (P2: *Lactobacillus acidophilus,* P3: *Lactiplantibacillus plantarum*) were recovered in this study although it was stated by the manufacturer to be a single species (*Lactobacillus acidophilus).* In a previous study, Anisimova et al*.* [52] recovered lactobacilli belonging to a single species from 2 dietary supplements despite being annotated by the manufacturer as multispecies. The detection of such discrepancies between the manufacturers’ claims and the actual products’ composition has become much easier thanks to the current progress in bacterial identification using more accurate and precise techniques like MALDI-TOF MS which has been demonstrated to be a rapid and reliable tool for the identification of lactobacilli to the species level [52, 53].

In the case of the strain P4, isolated from DSP4, its identification by MALDI-TOF MS was not opposing to that annotated by the manufacturer owing to the fact that both *L. casei* and *L. rhamnosus* are phenotypically and genotypically closely related species [54, 55].

Successful probiotic candidates are expected to have a good tolerance to the harsh conditions encountered in the GIT for the better survival of the probiotic strains to be able to exert their health benefits to the host. Such harsh conditions include the presence of high acidity, digestive enzymes, as well as bile [56]. All the studied LAB isolates, as well as the 2 reference LAB strains, were able to resist the acidic environment at pH 3 showing less than one log reduction in survivors compared to their initial count at 0 h. LAB have been reported to resist acid stress and adapt to a low pH environment through many resistance mechanisms such as the neutralization processes and the presence of H^+^-ATPase activity that helps to maintain the intracellular pH homeostasis through proton extrusion when present in an acidic environment [50]. Upon the addition of pepsin to the acidic medium at pH 3, both reference LAB strains and P3 showed no reduction in bacterial count, after 3 h, compared to the initial count at 0 h. The remaining isolates showed a slight reduction of bacterial count not exceeding 0.18 log reduction. Seah et al*.* have found that the viability of most of the tested *Lactobacillus* strains was enhanced by the addition of pepsin at low pH compared to their viability at acidic pH without pepsin. Pepsin is supposed to participate in promoting the function of H^+^-ATPase in bacterial protection and keeping pH homeostasis among LAB [57].

The viability of the tested strains was drastically reduced in the presence of pepsin at pH 2. Our findings were in concordance with previous studies where the survivability of the examined potential probiotic candidates was acceptable upon exposure to pH 3. However, a remarkable reduction in the viability was observed at lower pH (pH 2) [58–60]. Moreover, the probiotic strains are not likely to be directly subjected to the pH of the stomach as it could be buffered by food or other carrier matrix molecules following consumption [29]. This was obvious in our study where the examined strains showed better survivability upon the addition of RSM to pepsin at pH 2. Similarly, De Angelis et al*.* explained that the better resistance of the tested *Lactobacillus* strains to the simulated gastric juice at pH 2 was probably assigned to the rise in pH owing to RSM addition or the direct shielding activity exerted by the food matrix on bacteria [27].

Bile tolerance is one of the major selection criteria for potential probiotic strains where resistance to bile enables these strains to survive, colonize, and exert their beneficial action inside the GIT [29, 61]. In a previous study, Shokryazdan et al*.* showed that the degree of bile resistance among the tested potential probiotic candidates was strain-specific [56]. In the present study, all the tested strains showed good survival in the presence of 0.3% bile with log reduction in survivors ranging between 0.05 and 1. However, the viability of *L. acidophilus* isolate P2 was markedly reduced with more than 7 log reduction in survivors. Surprisingly, our findings pointed out that the LAB strains incorporated in dietary supplements that are already marketed as probiotic products may not ideally fulfill the stringent criteria of probiotics. Similar to our results, it was reported that the viability of the strains found in some tested probiotic supplements, among which were *L. acidophilus* strains, was reduced by approximately 50% in the presence of bile salts. In the same report, it was concluded that probiotics in some marketed supplements may exhibit poor acid and bile tolerance [12]. Previous studies demonstrated significant bile tolerance among *Lactobacillus* as well as *Pediococcus* strains [29, 62]. A common mechanism of bile resistance in LAB has been recommended to be strongly related to the presence of BSH activity which potentially exerts a detoxification action by catalyzing the hydrolysis of conjugated bile salts [63]. Among *Lactobacillus* spp., bile resistance might be attributed to alterations in the bacterial cell membrane and cell wall architecture in addition to the active efflux of bile salts [61].

Some studies also considered pancreatin tolerance as a prerequisite for probiotics efficacy [56, 64]. In the present study, all the tested LAB strains showed an acceptable tolerance to pancreatin (1.9 mg/mL) after 4 h incubation. Our results were in consonance with Ruiz-Moyano et al*.* who reported that 90.2% of the examined LAB strains survived after being exposed to pancreatic enzymes (1.9 mg/mL) for 3 h [65]. Moreover, Charteris et al*.* reported that the majority of the studied *Lactobacillus* strains exhibited a natural intrinsic resistance to simulated pancreatic juice with no viability reduction for up to 4 h [66].

Another crucial selection criterion for potential probiotic candidates is their adhesion ability to the intestinal epithelial cells which enables them to colonize in the GIT and establish their health benefits. CSH and autoaggregation properties are thought to be essential for the initial non-specific interaction between the bacterial cells and the intestinal mucosa [60, 67]. In our study, *L. acidophilus* P2 and *L. rhamnosus* P4 showed high percentages of CSH while the tested *P. acidilactici* P5 strain displayed the least hydrophobicity capacity. Similar to our findings, Colloca et al. have demonstrated the high hydrophobicity potential of *Lactobacillus* spp., among which were *L. acidophilus* and *L. rhamnosus* strains [68]*.* Additionally, a low hydrophobicity percentage (10.4%) of a studied *P. acidilactici* strain was reported by Xu et al*.* [69]. However, it should be noticed that low hydrophobicity of LAB strains will not necessarily mean poor adhesion to the intestinal mucosa where several studies reported that LAB isolates, among which were *Pediococcus* and *Lactobacillus* strains, can display high adhesion percentages to Caco-2 cells despite their poor CSH. Hence, it was concluded that CSH seemed to have little influence on bacterial adhesion [60, 70].

Bacterial autoaggregation may help in bacterial persistence and the achievement of the required probiotic benefit by the formation of an adequately large biomass inside the intestine [71]. Besides, the ability of a probiotic strain to autoaggregate on the intestinal mucosa creates a barrier, thus preventing pathogen colonization in the intestine [72]. In the present study, *L. acidophilus* P2 exhibited the highest percentage of autoaggregation (66%) among the studied LAB strains. Similarly, in a study conducted by Kos et al*.,* the potential probiotic strain *L. acidophilus* M92 showed a marked autoaggregation ability [73].

In a previous study, Divisekera et al*.* noticed that there was not one single collected isolate that exhibited all the agreeable probiotic properties [74]. Similarly, in our study, it was noticed that not all the desirable characteristics of probiotics existed within one isolate. Among the tested strains, P2 showed the least survival potential upon the addition of RSM to pepsin at pH 2, compared to other LAB strains. Also, it could not survive in the presence of 0.3% ox-bile. Since both criteria are essential for the survival of potential probiotic strains in the human GIT, P2 was excluded from the rest of the experiments. Among our isolates, 3 LAB strains from dietary supplements (*L. plantarum* P3, *L. rhamnosus* P4, and *P. acidilactici* P5), as well as the reference LAB strains, displayed superior probiotic capacities, and were included in the rest of the experiments for the in vitro characterization of the probiotic properties.

Phenols can be liberated in the gut as a result of the deamination process, caused by the gut bacteria, of different aromatic amino acids existing in dietary or endogenous proteins. The produced phenols exhibit a bacteriostatic action on the probiotic LAB. Hence, it is desirable for probiotic candidates to survive toxic metabolites in the GIT as phenols [75]. Phenol concentrations ranging between 0.2%-0.5% were selected in several published studies as the mean representative phenol content in phenol tolerance tests [51, 76]. In our study, all the tested LAB strains showed excellent growth at 0.2% phenol. However, their growth was markedly reduced with the increase in phenol concentration recording the least survival at 0.5% phenol. Similarly, it was previously reported that *L. plantarum, L. rhamnosus,* and *P. acidilactici* potential probiotic LAB strains showed about 90% relative growth on average at 0.2% phenol [33, 76]. Rahman et al*.* also reported that the examined probiotic candidates experienced poor growth at higher phenol concentrations (0.3% and 0.4%) [77]. Moreover, other studies demonstrated that *Lactobacillus* strains showed the least percentages of relative growth at 0.5% phenol [33, 75]. However, in our study, *P. acidilactici* P5 displayed the best survival, even at 0.5% phenol (percentage of relative growth of 39%), compared to the other tested LAB strains. In line with such finding, the potential probiotic strain belonging to *P. acidilactici* was previously reported to show growth, with a percentage of relative growth of ca. 65%, in the presence of high phenol concentration (0.5%) [76].

Despite the controversial opinions regarding the BSH activity exhibited by a probiotic strain, it can be considered a desirable property for the potential probiotic candidate as it can help in lowering the cholesterol level. Besides, it aids in the detoxification of bile salts thus promoting the survival of the producing strains in the intestine [29]. In our study, the tested *L. plantarum* probiotic strains (Ref 2, P3) and *P. acidilactici* (Ref 1, P5) were BSH-positive. On the other hand, *L. rhamnosus* P4 was BSH-negative. In agreement with our results, the presence of BSH activity in *L. plantarum* and *P. acidilactici* strains was previously reported [29, 62], while other studies did not detect BSH activity among *L. rhamnosus* strains [78, 79]. However, Shehata et al*.* previously detected weak BSH activity among *L. rhamnosus* strains [33]*.*

Among the major challenges facing the healthcare system are the emerging disaster of multi-drug resistant pathogens and the continuous fast decrease in antibiotics’ spectrum against such harmful bugs. Although probiotics cannot entirely replace the broadly used antibiotics, they might serve as valuable complements for antibiotics [80]. The antibiotic-probiotic coadministration might have the potential to overcome antimicrobial resistance and fight complex infections [81] mainly through providing synergized antimicrobial activity, thus, decreasing the required dose of an antibiotic, and contributing to intestinal flora replenishment [82].

One of the imperative selection criteria for probiotic strains is their antimicrobial activity against various pathogens [56]. Additionally, the safety assessment of any product intended for human use is the primary measure to be considered [83]. In our previous study, the antagonistic activity of the tested probiotic isolates P3, P4, and P5, as well as their CFS, against various indicator strains was investigated. Moreover, the evaluation of several safety aspects namely some virulence factors such as hemolytic, deoxyribonuclease, and gelatinase activities as well as the phenotypic and genotypic characterization of the antimicrobial resistance against some selected antibiotics was also done [84].

Recently, the world of microbial biotherapy has been directed towards the consumption of probiotic-derived components, like the CFS of probiotics, as a safer alternative to the use of the whole viable probiotic microorganisms [85].

In fact, the evaluation of the efficacy of metabiotic-antibiotic combinations against antibiotic-resistant clinical isolates has been recognized as a point of research of critical importance particularly in developing countries, especially Egypt, where escalating rates of antimicrobial resistance have been reported mainly due to the misuse of antibiotics.

As far as we know, there is a lack of research studies evaluating the combination of commercial dietary supplements with conventional antibiotics against antibiotic-resistant clinical isolates, using the time-kill assay, in Egypt. Therefore, investigating the efficacy of metabiotic-antibiotic combinations against isolates obtained from our country was a point of interest.

In the present study, the time-kill assay was used to assess the efficacy of in vitro combinations of the CFS of 2 probiotic candidates: *L. rhamnosus* P4*,* and *P. acidilactici* P5, as representatives from different genera, with each of the tested antibiotics (gentamicin, ciprofloxacin, and ceftazidime) against selected clinical isolates. Among the tested combinations, only 4 proved to have synergistic action against the examined pathogens. These synergistic combinations were the CFS of *L. rhamnosus* P4 / ceftazidime combination against *E. coli*^GIT^, the CFS of *L. rhamnosus* P4 / ceftazidime combination against *S. aureus*^UTI2^, the CFS of *L. rhamnosus* P4 / gentamicin combination against *E. coli*^GIT^, and the CFS of *P. acidilactici* P5 / ceftazidime combination against *S. aureus*^UTI2^. To our knowledge, the current literature lacks sufficient studies regarding the efficacy of combination therapy of probiotic strains isolated from commercially available dietary supplements and conventional antibiotics against *S. aureus* and *E. coli* clinical isolates using the time-kill assay. However, in a previous study, Singh et al*.* reported a synergistic effect for the combinations of CFS of *L. plantarum* with each of ampicillin, ciprofloxacin, and ceftriaxone, when tested using the time-kill assay, against *Salmonella typhimurium* [86]. In 2023, Lee et al. recorded that adding *Lactobacillus* spp. to combinations of two antibiotics showed synergistic antimicrobial activity against multidrug-resistant *Acinetobacter baumannii*, as revealed by time-kill assay [87]. In addition, some studies have reported the potential synergistic combinations of antibiotics and probiotic LAB strains against Gram-negative pathogens using other methods. Sharma et al*.* detected a marked enhancement in the inhibition zones of *E. coli* strains upon the combination of probiotic *Lactobacillus* strains, including *L. rhamnosus,* with each of ceftazidime and the aminoglycoside amikacin [88]. Biswas et al*.* have also reported the potential synergistic activity of the cephalosporin cefotaxime with the bacteriocin-producing probiotic strain *L. plantarum* against *E. coli* clinical isolates using Kirby-Bauer disc diffusion method [80]. Moreover, using the checkerboard method, other studies demonstrated promising synergistic combinations between the CFS of *Lactobacillus* strains, including *L. rhamnosus* and *L. plantarum,* and gentamicin against *P. aeruginosa* strains [10, 82].

It is worth mentioning that the difference in the efficacy of the tested metabiotic-antibiotic combinations among the studied clinical isolates might be attributed to strain variation as well as the different stress factors to which these isolates were exposed during the treatment period of the patients from which the isolates were obtained. Moreover, there is a possible variation in the potency of the tested combinations against isolates obtained from various geographical regions. Thus, testing the efficacy of such combinations against clinical isolates from Egypt is crucial to understanding the possible role of these combinations in combating antibiotic-resistant pathogens spreading in Egypt.

To elucidate the mechanism of the synergistic combination between antibiotics and probiotics, it was reported that the organic acids produced by probiotic strains could increase the permeability of the Gram-negative pathogen’s outer membrane besides lowering the pH. This may lead to the potentiation of the effects of other antimicrobial agents [89]. Moreover, H_2_O_2_, which can be secreted from probiotics, causes the peroxidation of membrane lipids and hence leads to the alteration in the permeability of the cell membrane. The combined action of H_2_O_2_ and aminoglycoside antibiotics to increase ROS resulted in an exaggerated bactericidal effect of the combination [10, 82]. It was previously reported that the activity of some β-lactams can be enhanced in the presence of acid media [90]. Also, MICs of β-lactams against *S. aureus* might decrease at acidic pH compared to neutral pH [91]. Besides, an increase in the binding of β-lactams to penicillin-binding proteins of bacteria at low pH was reported [92]. This could explain the synergized activity of ceftazidime with the organic acids produced by the probiotic candidates against the tested pathogens. Generally, bacteriocin/antimicrobial combinations can widen the spectrum of antimicrobial agents which may lead to a reduction in the antibiotic concentration required for efficient therapy, thus reducing, or even eliminating, the side effects [93].

On the contrary, in our study, no synergistic activity was detected among any of the combinations of ciprofloxacin with CFS of both probiotic candidates: *L. rhamnosus* P4 and *P. acidilactici* P5 against the tested clinical isolates. This could be attributed to the reduced activity of ciprofloxacin owing to the decline in pH of the medium exerted by the organic acids existing in the CFS of the tested probiotic strains. It has been previously reported that the antimicrobial activity of the old fluoroquinolone ciprofloxacin might be impaired by lowering the pH [94–96].

## Conclusions

Owing to the continuous rise in the rate of consumption of commercial probiotic dietary supplements, it has become a must to ensure that such products are capable of achieving the suggested health benefits for the consumers. Precise identification of LAB strains incorporated in dietary supplements is necessary where relying on advanced molecular or proteomic methods is recommended. LAB strains present in commercial probiotic supplements might not fulfill the stringent selection criteria for probiotics. Strict evaluation of the probiotic strains incorporated in dietary supplements, through further in vivo trials, is crucial to ensure the safety and efficacy of the probiotic candidates for human health benefits. Promising synergistic combinations of the CFS of selected LAB strains obtained from commercial probiotic supplements with conventional antibiotics could contribute to overcoming the antimicrobial resistance of problematic pathogens such as *E. coli* and *S. aureus.* Thus, the CFS of some probiotic candidates could be utilized for the formulation of novel biotherapeutic agents, as a safer alternative compared to the whole viable cells, for combating serious bacterial infections. However, a more comprehensive view of the auspicious outcomes of metabiotic-antibiotic combinations in solving the problem of antimicrobial resistance in developing countries should be provided. Further in vitro studies investigating the efficacy of CFS of a larger number of commercial probiotic strains from different species in combination with various conventional antibiotics against a wider pool of antibiotic-resistant isolates from Egypt are needed. In addition, future in vivo studies are required to evaluate the most appropriate treatment regimen for the CFS of each probiotic to control different microbial infections.

### Supplementary Information

Additional file 1: Time-kill assay of the CFS of *L*. *rhamnosus* P4 and ceftazidime, each alone and in combination, against *E*. *coli*^UTI^.

Additional file 2: Time-kill assay of the CFS of *L*. *rhamnosus* P4 and gentamicin, each alone and in combination, against (a) *E*. *coli*^UTI^ and (b) *S*. *aureus*^UTI2^.

Additional file 3: Time-kill assay of the CFS of *L*. *rhamnosus* P4 and ciprofloxacin, each alone and in combination, against (a) *E*. *coli*^UTI^, (b) *E*. *coli*^GIT^ and (c) *S*. *aureus*^UTI2^.

Additional file 4: Time-kill assay of the CFS of *P*. *acidilactici* P5 and ceftazidime, each alone and in combination, against (a) *E*. *coli*^UTI^ and (b) *E*. *coli*^GIT^.

Additional file 5: Time-kill assay of the CFS of *P*. *acidilactici* P5 and gentamicin, each alone and in combination, against (a) *E*. *coli*^UTI^, (b) *E*. *coli*^GIT^ and (c) *S*. *aureus*^UTI2^.

Additional file 6: Time-kill assay of the CFS of *P*. *acidilactici* P5 and ciprofloxacin, each alone and in combination, against (a) *E*. *coli*^UTI^, (b) *E*. *coli*^GIT^ and (c) *S*. *aureus*^UTI2^.

## Data Availability

Most data generated or analysed during this study are included in this published article and its additional files. Any extra demanded details are available from the corresponding author on reasonable request.

## Abbreviations

CFS: Cell-free supernatants
LAB: Lactic acid bacteria
ATCC: American Type Culture Collection
MRS: De Man, Rogosa, Sharpe
MALDI-TOF MS: Matrix-assisted laser desorption ionization time-of-flight mass spectrometry
GIT: Gastrointestinal tract
PBS: Phosphate-buffered saline
RSM: Reconstituted skimmed milk
CSH: Cell surface hydrophobicity
BSH: Bile salt hydrolysis
MIC: Minimum inhibitory concentration
