# Supplementary figures and images for: Characterization of probiotics isolated from dietary supplements and evaluation of metabiotic-antibiotic combinations as promising therapeutic options against antibiotic-resistant pathogens using time-kill assay
Source: BMC Complement Med Ther. 2024 Aug 14;24:303. doi: 10.1186/s12906-024-04582-3 (PMC11325838; doi:10.1186/s12906-024-04582-3)

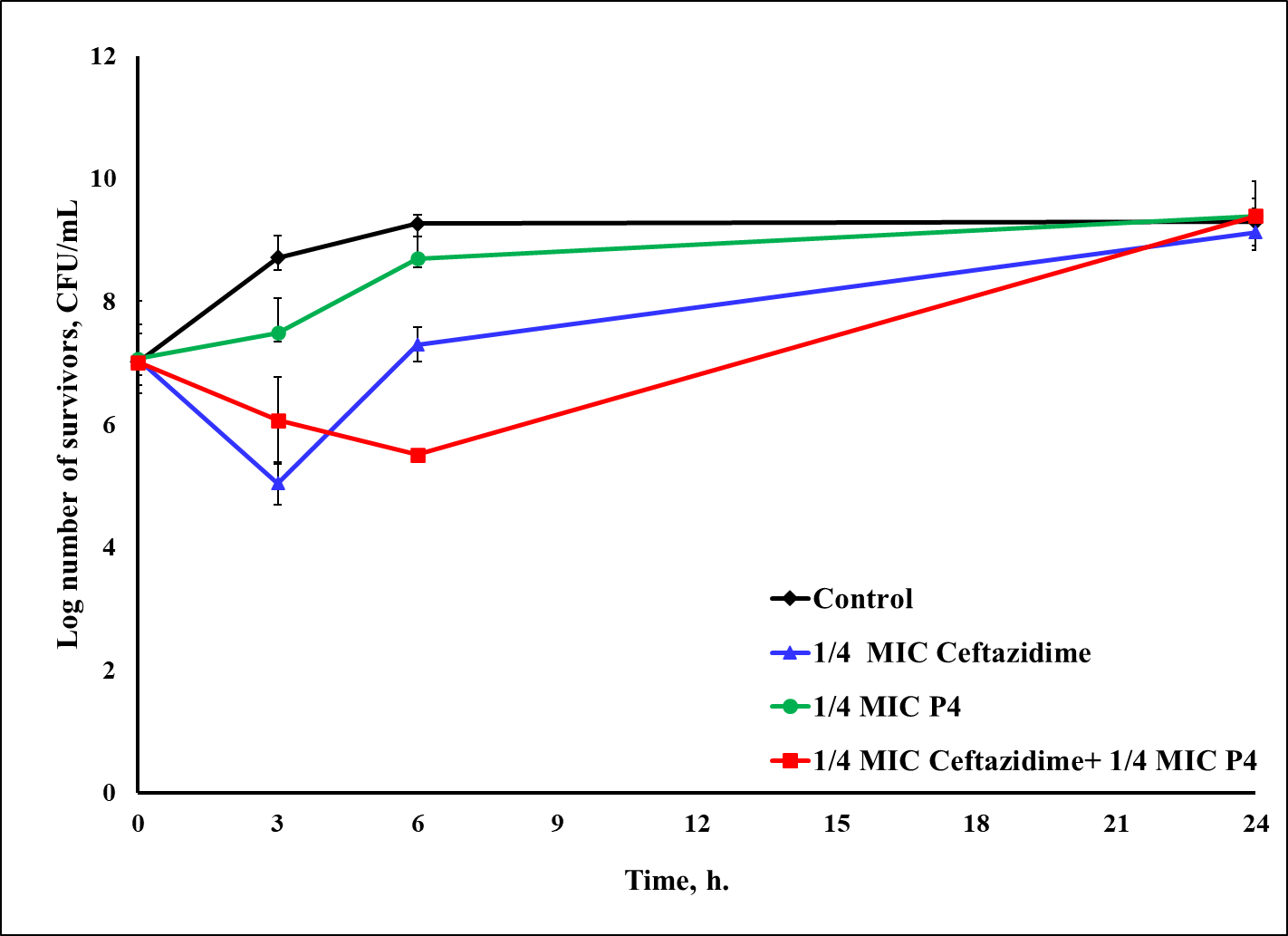

Supplement: Supplementary file 1 — Additional file 1: Time-kill assay of the CFS of L. rhamnosus P4 and ceftazidime, each alone and in combination, against E. coliUTI. [file 12906_2024_4582_MOESM1_ESM.tif]

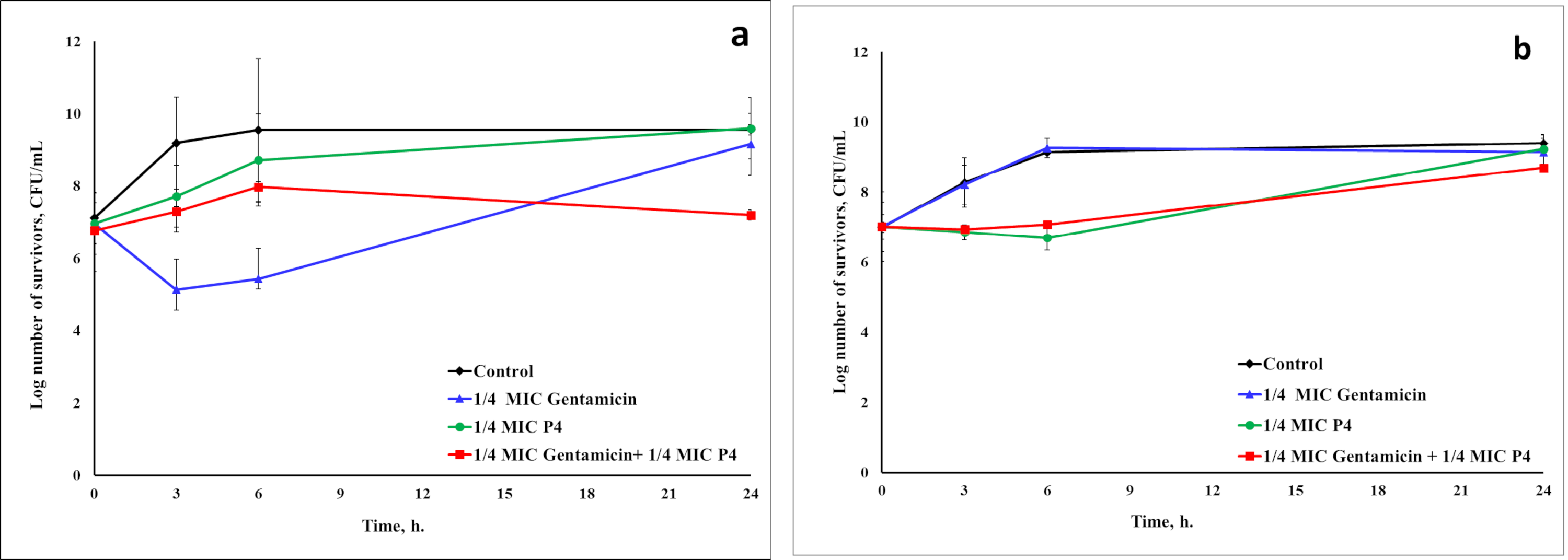

Supplement: Supplementary file 2 — Additional file 2: Time-kill assay of the CFS of L. rhamnosus P4 and gentamicin, each alone and in combination, against (a) E. coliUTI and (b) S. aureusUTI2. [file 12906_2024_4582_MOESM2_ESM.tif]

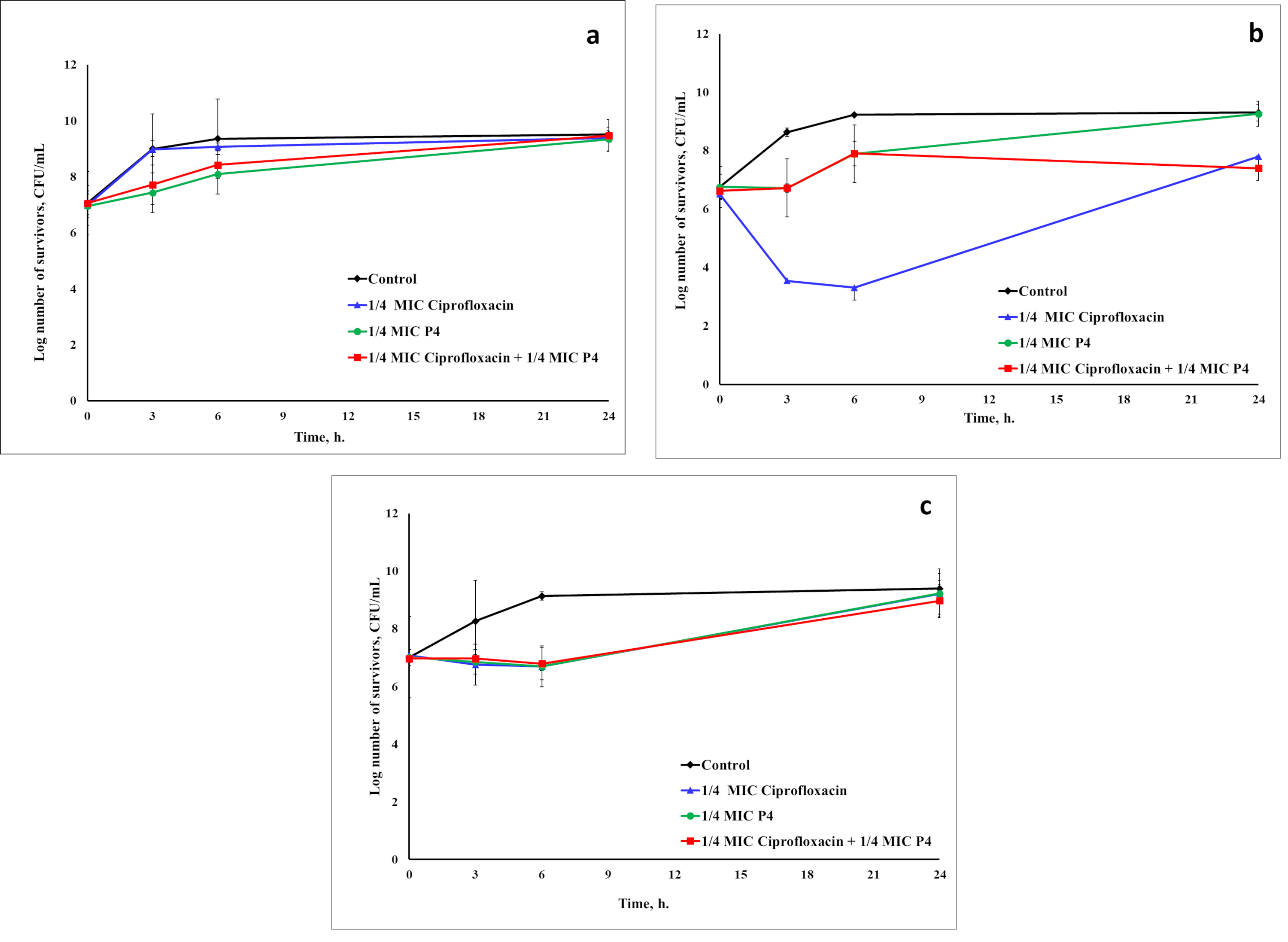

Supplement: Supplementary file 3 — Additional file 3: Time-kill assay of the CFS of L. rhamnosus P4 and ciprofloxacin, each alone and in combination, against (a) E. coliUTI, (b) E. coliGIT and (c) S. aureusUTI2. [file 12906_2024_4582_MOESM3_ESM.tif]

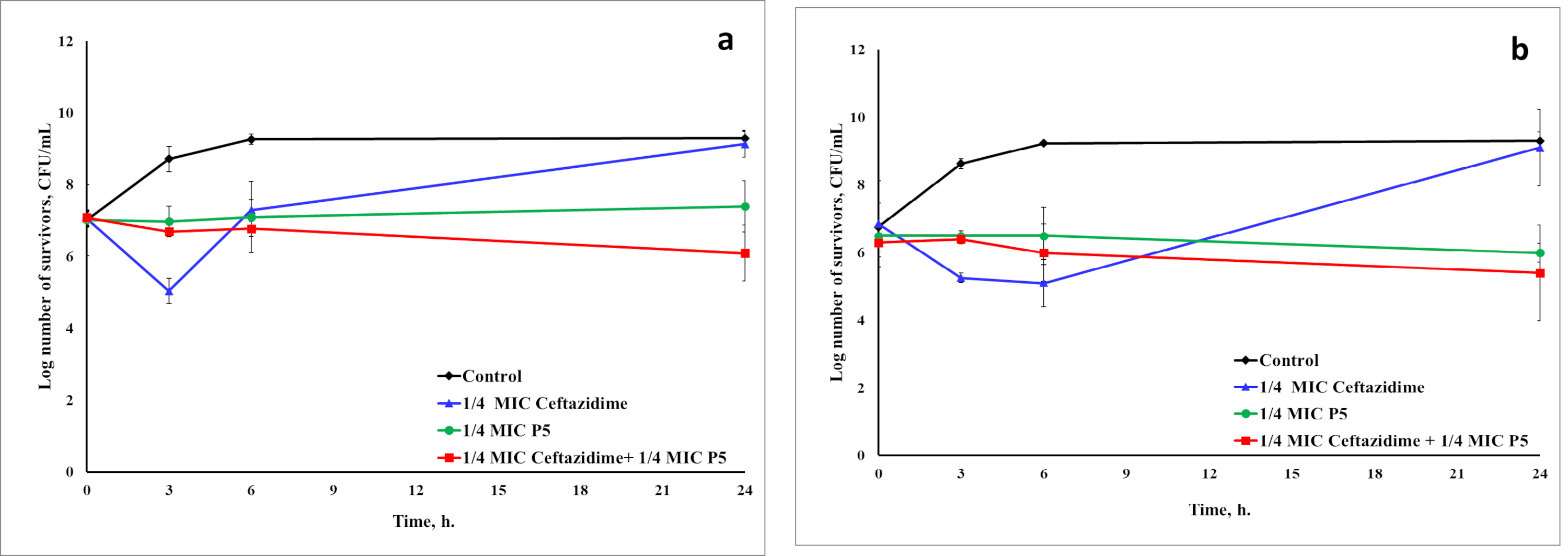

Supplement: Supplementary file 4 — Additional file 4: Time-kill assay of the CFS of P. acidilactici P5 and ceftazidime, each alone and in combination, against (a) E. coliUTI and (b) E. coliGIT. [file 12906_2024_4582_MOESM4_ESM.tif]

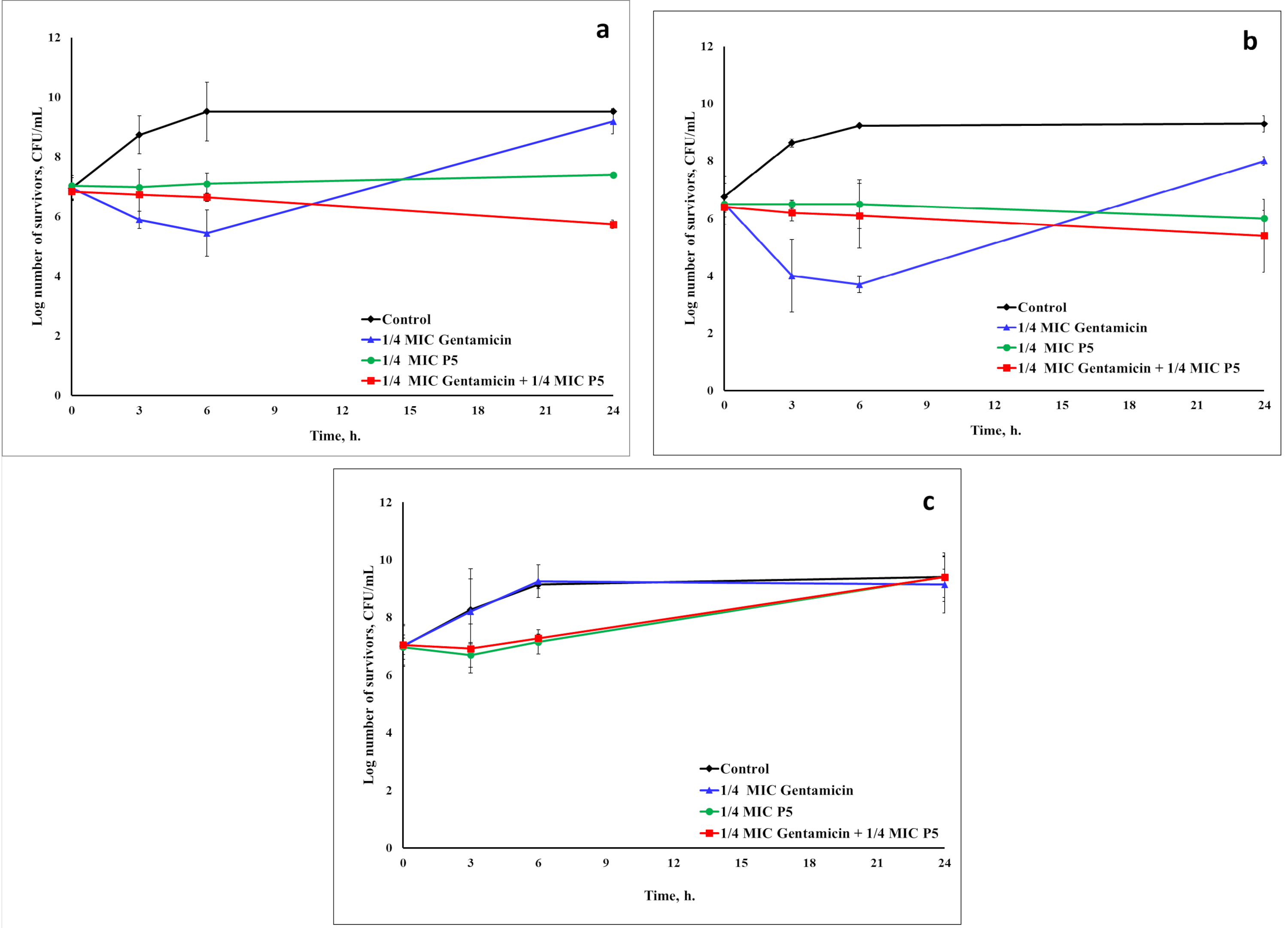

Supplement: Supplementary file 5 — Additional file 5: Time-kill assay of the CFS of P. acidilactici P5 and gentamicin, each alone and in combination, against (a) E. coliUTI, (b) E. coliGIT and (c) S. aureusUTI2. [file 12906_2024_4582_MOESM5_ESM.tif]

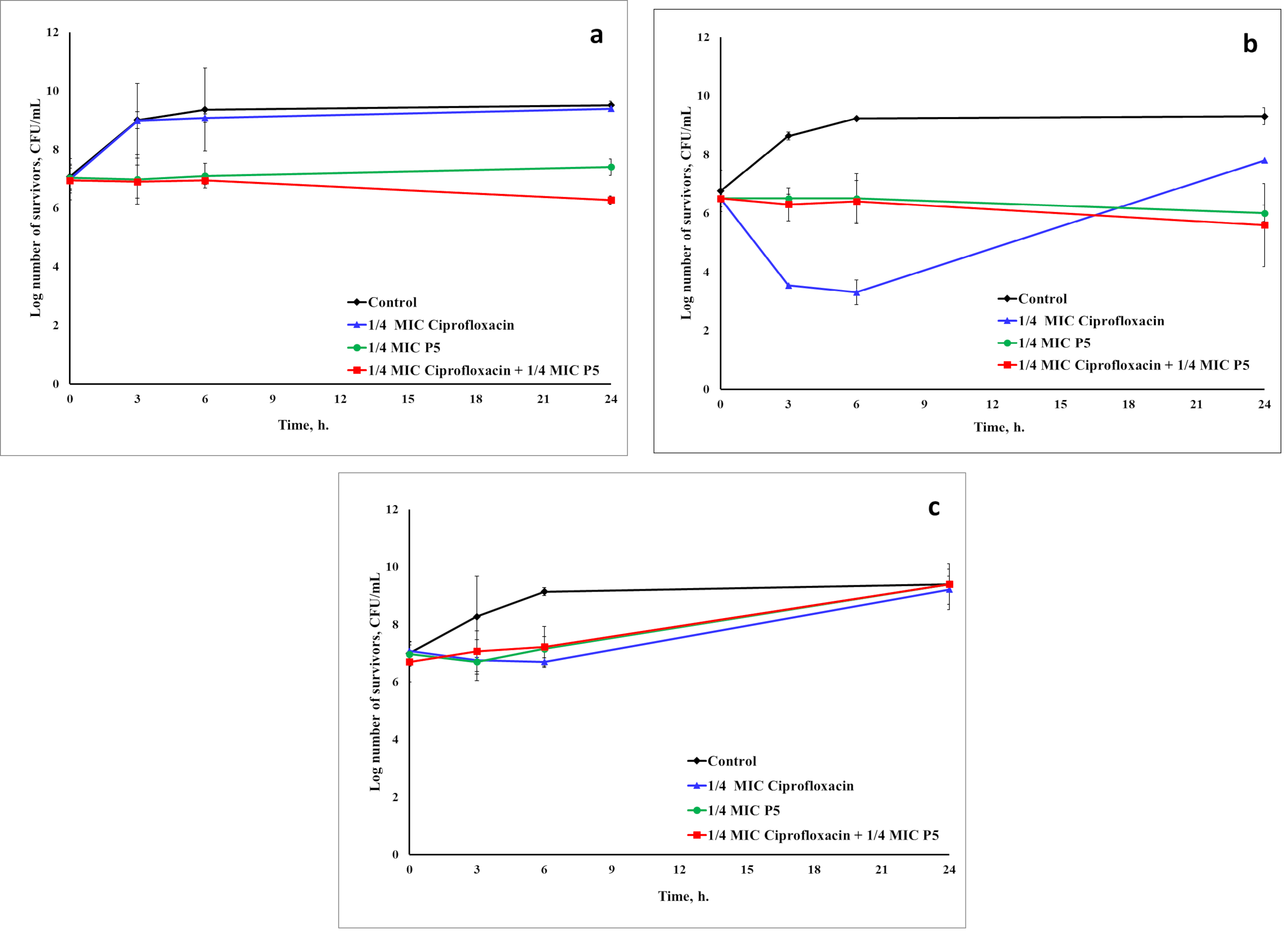

Supplement: Supplementary file 6 — Additional file 6: Time-kill assay of the CFS of P. acidilactici P5 and ciprofloxacin, each alone and in combination, against (a) E. coliUTI, (b) E. coliGIT and (c) S. aureusUTI2. [file 12906_2024_4582_MOESM6_ESM.tif]
